# Anxiety and Depression Assessments in a Mouse Model of Congenital Blindness

**DOI:** 10.3389/fnins.2021.807434

**Published:** 2022-01-21

**Authors:** Nouhaila Bouguiyoud, Florence Roullet, Gilles Bronchti, Johannes Frasnelli, Syrina Al Aïn

**Affiliations:** ^1^Department of Anatomy, Université du Québec à Trois-Rivières, Trois-Rivières, QC, Canada; ^2^Cognition, Neurosciences, Affect et Comportement (CogNAC) Research Group, Université du Québec à Trois-Rivières, Trois-Rivières, QC, Canada; ^3^Department of Psychiatry and Behavioural Neurosciences, McMaster University, Hamilton, ON, Canada

**Keywords:** depression, anxiety, congenital blindness, mice, exploration

## Abstract

Previous studies have reported that visual impairment can affect the quality of life leading to mental health disorders. This study aimed to investigate associations between vision impairment, depression and anxiety using a mouse model of congenital blindness. We phenotyped 15 anophthalmic and 17 sighted adult mice in a battery of tests for anxiety and depression-like behaviors: open field test, elevated plus maze, coated test, splash test, and forced swim test. We found that: (1) Anxiety levels of the anophthalmic mice were significantly lower when compared with sighted mice, (2) Anophthalmic mice displayed more exploratory behaviors in a new environment than the sighted one, and (3) Depression levels between those groups were similar. In conclusion, this behavioral study showed that early visual deprivation lowers anxiety levels, associated with heightened exploratory activity, but does not induce depressive symptoms in a mouse model of congenital blindness, underlying several behavioral adaptations.

## Introduction

In 2020, the number of people with blindness and with severe or moderate vision impairment (VI) was estimated to, 43.3 million and 295 million, respectively. Blindness and VI affect the quality of life, social activities, daily occupations and are considered as vulnerability factors of mental health disorders, such as depression and anxiety (Nyman et al., 2010; van der Aa et al., 2015; Demmin and Silverstein, 2020). Systematic reviews reported that the prevalence of depressive symptoms in visually impaired individuals varies between 14 and 44% (Horowitz et al., 2005; van der Aa et al., 2015; Osaba et al., 2019), although most of these studies did not distinguish etiology, age of onset and duration of the visual impairment. Prenatal and perinatal causes, such as genetic factors, perinatal complications, dysfunctions in embryonic development, central nervous system alteration may lead to congenital visual deprivation. Degenerative disorders, trauma, and infectious diseases of the eye may induce sudden or progressive vision loss after birth and are defined as adventitious visual deprivation (West and Sommer, 2001; Demir et al., 2014). Depression in older visually impaired people seems to be a common problem (Horowitz et al., 2005; Mathew et al., 2011; Renaud and Bédard, 2013). Additionally, few studies have found increased rates of anxiety among visually impaired populations (Demmin and Silverstein, 2020, for review).

Less is known about the presence of depression and anxiety in people with early blindness and the findings appear scarce and inconsistent. Interestingly, one study conducted on forty congenitally blind children and teenagers aged 11–14 years found significantly lower levels of depression and anxiety, less behavioral total problems scores, as well as lower attention problems subscales scores compared to forty sighted children and teenagers (Demir et al., 2014). Other studies looking at teenagers with congenital and early blindness (caused by retinopathy of prematurity, congenital cataract, juvenile hereditary retinoschisis, albinism, or optic atrophy) found no difference in depression scores (Huurre and Aro, 1998; Bolat et al., 2011), but higher anxiety levels (Bolat et al., 2011). However, Koenes and Karshmer (2000) reported a greater prevalence of depression in congenitally blind adolescents compared to sighted adolescents. In addition, few behavioral studies on genetic models of early blind animals showed a similar level of anxiety in early blind adults and their sighted counterparts (Buhot et al., 2001; Iura and Udo, 2014). To the best of our knowledge, no study investigated the effect of early visual deprivation on depression in animal models.

This study aimed to explore depression- and anxiety-like symptoms in a mouse model of congenital blindness, the ZRDBA mouse strain. The ZRDBA strain has been used as a model of congenital visual deprivation obtained by crossbreeding between two strains: the anophthalmic ZRDCT and the sighted DBA-6 strains (Touj et al., 2019). The anophthalmic ZRDCT strain described first by Chase and Chase (1941), has a mutation on chromosome 18 on the Rx/Rax gene, which causes the absence of the eyes, the optic tracts, and the afferents retina-hypothalamus. The crossbreeding results in a litter with an equal number of anophtalmic Rx/Rax homozygous and sighted Rx/Rax heterozygous pups. Depression-like behaviors were assessed using the Coated (fur quality), splash (sucrose), Forced Swim and Suspension Tail tests. Anxiety-like behaviors were assessed in the Open Field and the Elevated Plus-Maze tests.

## Methods

### ZRDBA Mice

We tested a total of 32 adult mice aged 3–4 months old, including 15 blind (5 males and 10 females) and 17 sighted blind (5 males and 12 females) mice. All mice were bred and housed, in the animal facility at our institution, in groups (4–6 animals/cage) under standard environmental conditions of 12/12 h light/dark cycle (light phase: 7:00–19:00 h) at a controlled room temperature (20–22°C and 40–60% humidity) and food and water were provided *ad libitum*. Experimental procedures and animal use were permitted by the animal care committee of the Université du Québec à Trois-Rivières, in accordance with the guidelines of the Canadian Council on Animal Care.

### Behavioral Procedures

Behavioral experiments were performed under dim light conditions during the light phase between 9:00 a.m. and 5:00 p.m. A battery of anxiety-like and depression-like behavioral tests was conducted in the following order: Open-field test (OFT) (day 1), Coated test (day 1), Splash test (day 1), Elevated-plus maze (EPM) (day 3), Forced Swim Test (FST) (day 6), and Tail suspension test (TST) (day 9). All the recordings and coding were done using Ethovision XT software (Noldus, Virgina, USA).

#### Depression-Like Behaviors

##### Coated Test

The protocol was adapted from Isingrini et al. (2010). This test assesses decreased self-care which is defined as depression-like behavior. The quality of the fur from the whole body divided into 6 regions (such as the head, neck, back, abdomen, forepaws, and hind paws) was scored as follows: 0 (well-groomed), 0.5 (moderately groomed), or 1 (unkept) were attributed for each area, for a maximum of 6 points (6 regions) per animal.

##### Splash Test

The protocol, adapted from Isingrini et al. (2010), consists in spraying a 10% sucrose solution on the dorsal coat of a mouse. Latency (time between spray and initiation of grooming), frequency and duration of grooming, and total distance traveled were recorded for 5 min and manually coded as an index of self-care and motivational behavior. Reduced grooming behavior is considered as a depression-like behavior of mice.

##### Forced Swim Test (FST)

The protocol was adapted from Can et al. (2012a). Mice were placed in an inescapable glass (height 21 cm) cylinder filled with 15 cm of water. Three types of behavior were videotaped for 6 min and analyzed: (1) swimming behavior was defined as horizontal movement through the water-filled cylinder, (2) climbing behavior referred to upward-directed movements of the forepaws along the wall of the water-filled cylinder, and (3) immobility consisted in floating with limited or no movements to maintain equilibrium.

##### Tail Suspension Test (TST)

The protocol was adapted from Can et al. (2012b). Mouse was suspended 50 cm above the floor in a tail suspension box (55 height × 60 width × 11.5 cm depth) by an adhesive tape placed 1 cm from the tip of the tail. To prevent any tail climbing behavior during the test, a plastic tubing (4 cm length × 1 cm diameter) was placed around the tails of mice. The time spent immobile in the TST was recorded for 6 min.

#### Anxiety-Like Behaviors

The open-field test and the elevated-plus maze are the most usual, validated behavioral tests assessing anxiety-like behaviors. Those devices are stressful environments for both blind and sighted mice given that devices are unfamiliar, without any bedding and hidden place except walls and enclosed arms. Therefore, open-field and EPM tests refer to the animal's aversion to open spaces and tendency to be thigmotaxic based on visual and non-visual cues. The anxious response is expressed by the animal spending more time in the enclosed arms or close to the walls.

##### Open Field Test (OFT)

The protocol was adapted from Seibenhener and Wooten (2015). The open-field device consisted of a square box (34 cm × 34 cm) with surrounding walls (height 49.5 cm) in Plexiglas, with opaque walls that avoid any visual cue bias. The center of the arena was delimited as the central 24 cm × 24 cm zone. Exploratory activities in the open field were recorded for 10 min by analyzing: (1) the total time spent in the center zone (as a measure of anxiety-like behavior), (2) the time spent and frequency of grooming (as a measure of anxiety-like behavior), (3) the total distance traveled (exploratory behavior), and (4) the time spent rearing (as a measure of exploratory behavior).

##### Elevated Plus Maze (EPM)

The protocol was adapted from Rodgers and Cole (1995). The elevated plus maze consisted of two arms without walls (elevated unprotected area) and two enclosed (elevated protected area) by walls (5 cm width × 29 cm length × 11.3 cm height), with a center area of 5 cm × 5 cm. Mice were placed into the center area and the duration and frequencies of entries in the open and closed arms and total distance traveled were coded manually. The test lasted 5 min.

#### Data Analysis

Statistical analyses were performed using SPSS software ver. 22.0 (IBM, Armonk, NY, USA). All data are expressed as mean ± SE. The distribution of our data was examined for normality using the Shapiro Wilk test. For each behavioral test, blind and sighted mice were compared using a multivariate analysis of variance (two independent factors: 2 visual groups and 2 sex status, dependent variables: measures within a test) for normally distributed data. Mann-Whitney tests were performed for non-normal data. Correlations between variables were performed using Spearman's correlation (for non-parametric data). For all analyses, the significance level was set at *p* < 0.05; with appropriate control (Bonferroni *post-hoc*) for multiple comparisons.

## Results

Analysis of data using MANOVA with sex as a factor did not result in any effect of sex or interaction between sex and vision (*data not shown*).

### Depression-Like Behaviors

**Figure 1:**
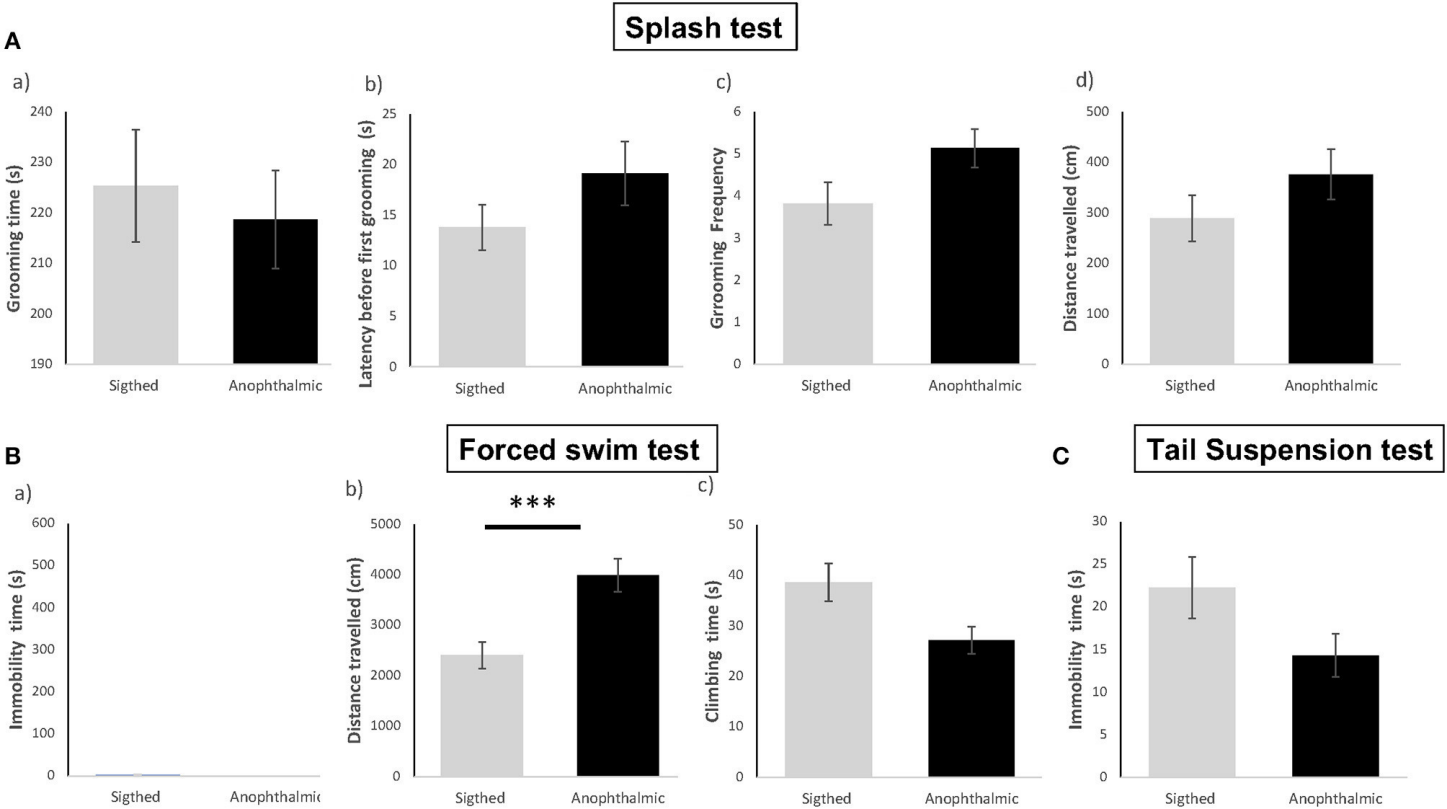
Anophthalmic and sighted mice assayed to depression-like behavioral tests, such as the Splash Test **(A)** : Mean (± SE) of (a) time spent (s) Grooming, (b) Latency (s) before the first grooming, (c) Grooming Frequency, (d) Distance traveled (cm) in the device for a 10 min-test; and the Forced Swim Test **(B)**: (a) Immobility Time (s), (b) Distance traveled (cm), (c) Time spent climbing in the tank for a 6-min test, and the Suspension Tail test **(C)**: Immobility Time (s) for a 6-min test (*N* = 15 and 17 mice/group, respectively ^***^p < 0.001).

All data are illustrated in Figure 1.

#### Coated Test

All of the blind and sighted mice obtained a score of 0 for each body area (*data not shown*).

#### Splash Test

In the splash test, both groups showed no significant difference in time spent grooming [*F*_(1,30)_ = 0.198, *p* > 0.05], latency to groom [*F*_(1,30)_ = 1.954, *p* > 0.05], frequency of grooming [*F*_(1,30)_ = 3.591, *p* > 0.05] and total distance traveled [*F*_(1,30)_ = 1.652, *p* > 0.05].

#### Forced Swim Test

In the Forced Swim Test, overall, most mice from both groups remained active and showed no immobility time throughout the 6-min test. Data for total immobility time and total distance did not pass the Shapiro-Wilk normality test (*W* = 0.308, *p* < 0.001 and *W* = 0.890, *p* < 0.005, respectively), hence statistical analysis was performed using Mann Whitney *U* non-parametric test. Both groups showed no significant difference in immobility time (*U* = 105, *p* > 0.05), time spent climbing [*F*_(1,30)_ = 5.944, *p* > 0.05] and total distance traveled (*U* = 233, *p* = 0.001).

#### Suspension Tail Test

Data for total immobility time were not normally distributed (*W* = 0.898; *p* = 0.007). No significant difference in immobility time was demonstrated between both groups (*U* = 72.5, *p* > 0.05).

### Anxiety-Like Behaviors

**Figure 2:**
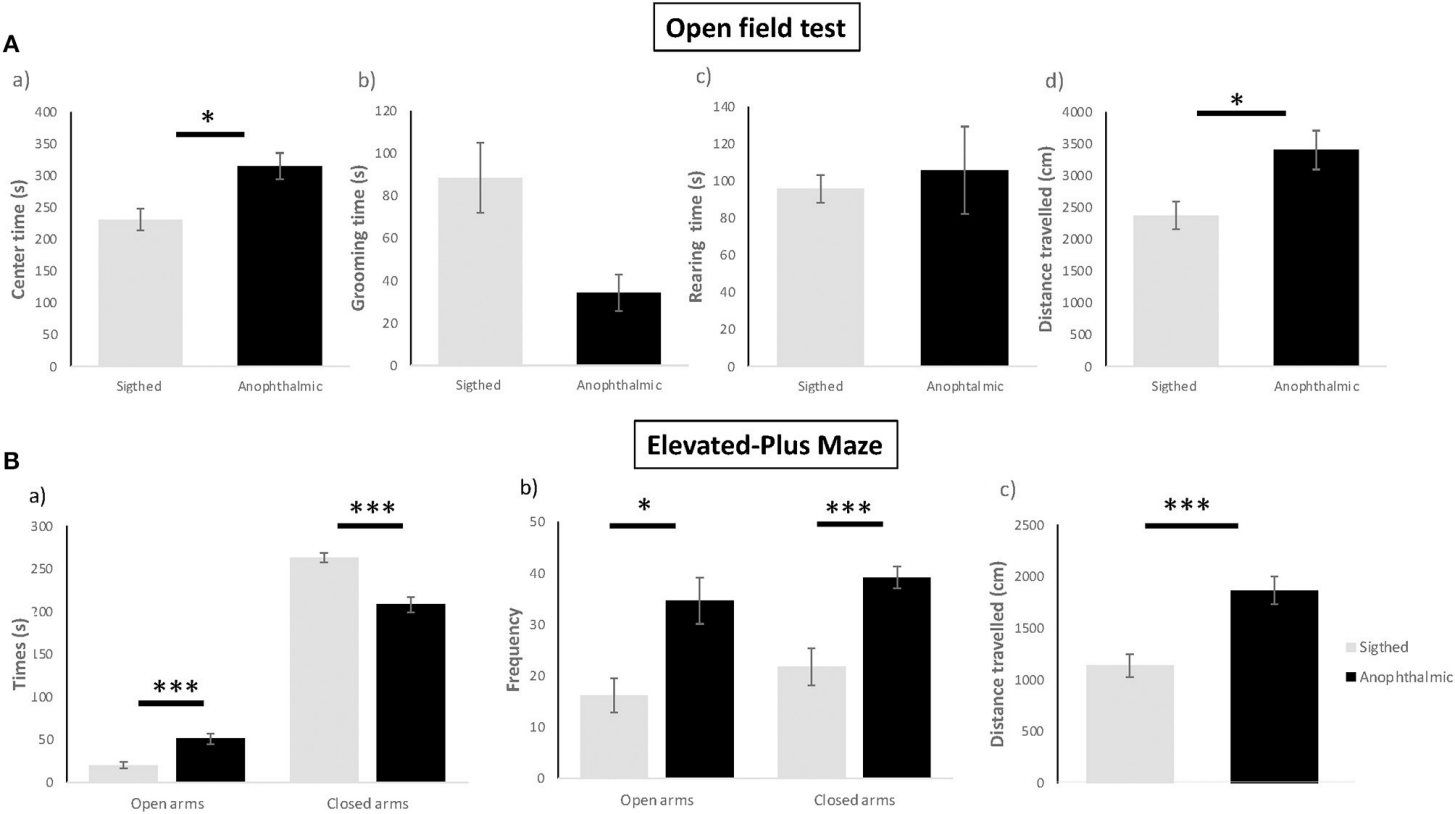
Anxiety-like behavioral tests. Anophthalmic and sighted mice assayed to the open-field test **(A)**: (a) Mean (± SE) time spent (s) of grooming, (b) Latency before the first grooming (s), (c) Frequency of grooming, (d) Distance (cm) traveled in the device for a 5 min-test (*N* = 15 and 17 mice/group, respectively). Anophthalmic and sighted mice assayed to the Elevated-Plus Maze **(B)**: (a) Mean (± SE) time spent (s) in open and closed arms, (b) Frequency in open and closed arms, (c) Distance (cm) traveled in the whole device for a 5 min-test (*N* = 15 and 17 mice/group, respectively; *p < 0.05; ***p < 0.001).

All data were illustrated in Figure 2.

#### Open Field Test

Blind mice exhibited a higher total time spent in the center zone [*F*_(1,30)_ = 9.943, *p* = 0.012], an increased total distance traveled in the arena [*F*_(1,30)_ = 7.86, *p* = 0.027], as compared to sighted mice. No significant differences were shown for the time spent grooming (*U* = 67, *p* > 0.05) and rearing between both groups [*F*_(1,30)_ = 0.184, *p* > 0.05].

#### Elevated Plus Maze

Blind mice spent more time in the open arms [*F*_(1,28)_ = 17.651, *p* < 0.001], spent less time in the closed arms [*F*_(1,28)_ = 26,470, *p* < 0.001], displayed higher frequency of entries in the open arms [*F*_(1,28)_ = 10.496, *p* = 0.012] and lower frequency of entries in the closed arms [*F*_(1,28)_ = 15,535, *p* < 0.001] than sighted mice. In addition, blind mice exhibited a higher distance traveled overall [*F*_(1,28)_ = 16.206, *p* < 0.001] than sighted mice.

### Correlations

Spearman's correlations are shown in Table 1. No association was demonstrated between indexes of anxiety, such as time spent in the center area in the OFT and the open arms in the EPM (*p* > 0.05), but a negative correlation was found between the time spent in the center area and the time grooming in the OFT [*r*_(32)_ = −0.493, *p* = 0.004], indicating that mice who explored more unprotected area, spent less time grooming accordingly in the OFT. Regarding depression indexes, such as the time spent grooming in the Splash test, immobility time in the FST and the TST, were not correlated between them (*p* > 0.05).

**Table 1:** Spearman's correlations amongst behavioral variables assessing depression-like and anxiety-like behaviors.

**Spearman's correlations (Rho)**	**SPL TimeGroom**	**SPL LatGroom**	**SPL FreqGroom**	**SPL Distance**	**FST Distance**	**OFT Center**	**OFT Groom**	**OFT Distance**	**EPM TimeOA**	**EMP FreqOA**	**EMP TimeCA**	**EMP FreqCA**
**SPL_Distance**	−0.653**	0.350*	0.405*									
Sig. (bilateral)	<0.001	0.05	0.021									
**TST_Immo**	0.089	−0.048	−0.022	−0.067	−0.423*							
Sig. (bilateral)	0.64	0.799	0.908	0.724	0.02							
**OFT_Center**	−0.096	0.241	0.544**	0.434*	0.163							
Sig. (bilateral)	0.601	0.184	0.001	0.013	0.372							
**OFT_Groom**	0.321	0.096	−0.492**	−0.26	−0.022	−0.493**						
Sig. (bilateral)	0.073	0.6	0.004	0.151	0.906	0.004						
**OFT_Distance**	0.043	0.058	0.463**	0.013	0.236	0.25	−0.501**					
Sig. (bilateral)	0.817	0.753	0.008	0.943	0.193	0.168	0.004					
**OFT_Rear**	0.115	−0.194	−0.178	−0.032	−0.132	−0.084	0.056	−0.417*				
Sig. (bilateral)	0.53	0.286	0.329	0.86	0.472	0.648	0.759	0.017				
**EPM_TimeOA**	−0.035	0.32	0.062	0.19	0.616**	0.269	−0.23	0.158				
Sig. (bilateral)	0.855	0.085	0.743	0.314	<0.001	0.15	0.222	0.405				
**EMP_FreqOA**	−0.142	0.179	0.131	0.213	0.520**	0.221	−0.142	0.10	0.796**			
Sig. (bilateral)	0.453	0.345	0.489	0.258	0.003	0.24	0.453	0.598	<0.001			
**EMP_TimeCA**	0.109	−0.264	−0.179	−0.21	−0.694**	−0.358	0.339	−0.265	−0.943**	−0.811**		
Sig. (bilateral)	0.566	0.159	0.343	0.264	<0.001	0.052	0.067	0.157	<0.001	<0.001		
**EMP_FreqCA**	−0.322	0.237	0.312	0.375*	0.500**	0.426*	−0.343	0.265	0.721**	0.808**	−0.790**	
Sig. (bilateral)	0.083	0.207	0.094	0.041	0.005	0.019	0.064	0.158	<0.001	<0.001	<0.001	
**EMP_Distance**	−0.061	0.265	0.144	0.289	0.579**	0.366*	−0.26	0.174	0.852**	0.881**	−0.864**	0.824**
Sig. (bilateral)	0.75	0.157	0.448	0.121	<0.001	0.047	0.166	0.357	<0.001	<0.001	<0.001	<0.001
												

In the Splash Test: SPL_TimeGroom, grooming time; SPL_LatGroom, Latency before the first grooming; SPL_FreqGroom, frequency of grooming; SPL_Distance: total distance traveled. In the FST: FST_Distance, total distance traveled. In the TST: TST_Immo, Immobility time; OFT_Center, time spent in the center area; OFT_Groom, grooming time; OFT_Distance, total distance traveled; OFT_Rear, Rearing time. In the EPM: EPM_TimeOA, time spent in the open arms; EMP_FreqOA, frequency of entries in the open arms; EMP_TimeCA, time spent in the closed arms; EMP_FreqCA, frequency of entries in the closed arms; EMP_Distance, total distance traveled. Significant values were indicated in bold (^**^p < 0.01, ^*^p < 0.05).

#### Anxiety and Depression

In addition, increased time spent in the center area and reduced grooming time in the OFT were associated with high frequency to groom in the Splash test [*r*_(32)_ = 0.544, *p* = 0.001; *r*_(32)_ = −0.492, *p* = 0.004], indicating that lower is the level of anxiety, lower is that of depression.

#### Anxiety and Exploration

Time spent in open area in the OFT was positively correlated with distance traveled in the EPM [*r*_(32)_ = 0.366, *p* = 0.047] and distance traveled in the Splash test [*r*_(32)_ = 0.434, *p* = 0.013]. The time spent grooming in the OFT is negatively associated with distance traveled in the OFT [*r*_(32)_ = −0.501, *p* = 0.004]. This underlies that low anxiety-related behaviors were associated with an increased locomotor activity.

#### Depression and Exploration

High frequency to groom in the Splash test (low depression) was also associated with heightened distance traveled in the OFT [*r*_(32)_ = 0.463, *p* = 0.008]. Time spent immobile in TST (low depression) was negatively correlated with distance traveled in the FST [*r*_(32)_ = −0.423, *p* = 0.02], suggesting that low depression-like behaviors were associated with an increased locomotor activity.

## Discussion

Here, we showed that congenitally blind mice exhibit less anxiety-like behavior than sighted controls. In contrast, for depression-like behavior, there was no difference between both groups. Interestingly, early visual deprivation resulted in increased locomotor activity levels. Regarding correlation analysis, we pointed out that indexes of depression were not correlated between them, just like indexes of anxiety. Importantly, this study confirms an association between all the three factors, anxiety-like and depression-like behaviors and exploratory activity: Low levels of anxiety-like and depression-like behaviors are associated with heightened exploration drive.

No differences were observed in the Coated, Splash, Forced Swim and Tail suspension tests between both groups of mice. In the Forced Swim test, both sighted and blind ZRDBA mice showed no immobility for the entire duration of the test (6 min long). This lack of immobility at baseline in the FST has been reported previously for certain strains of mice such as the Black Swiss and FVB/NJ (Can et al., 2012a) and may indicate that the test might not be the most appropriate for our strain. It is worthy to note that retinal degeneration has been observed in Black Swiss mouse eyes, which causes visual impairment in adulthood (Clapcote et al., 2005). Whereas, depression is a significant issue in visually impaired older adults (for review Demmin and Silverstein, 2020), the link between early blindness and mental health has been well-described yet remains unclear. Indeed, when examining the behavioral outcomes in humans, observations vary: Adolescents with congenital blindness present lower levels of depression, anxiety and withdrawal behaviors as compared to sighted adolescents (Demir et al., 2014) whereas higher levels of anxiety, but not depression, were found in adolescents with visual impairment compared to sighted adolescents (Bolat et al., 2011). Consistent with previous studies (Buhot et al., 2001; Iura and Udo, 2014), we reported that blind and sighted mice showed similar behaviors in thigmotaxis, which refers to the tendency to touch the walls with actual physical contact, such as closed, protected arms, compared to exposed, unsafe areas in the EPM. Whereas, lower anxious behavior was evidenced in the OFT and the EPM tests in our blind mice, other studies found that anophthalmic mice displayed the same level of anxiety than sighted ones in the EPM (Buhot et al., 2001; Iura and Udo, 2014). Even though blind mice did not exhibit anxiety induced by brightness or height perceptions, they preferred closed arms in the elevated plus maze test. In other words, blind mice, just like sighted ones, exhibited an innate fear from open spaces. Despite visual deprivation, blind mice did not drop from the narrow platform in the EPM. These findings indicate that blind mice have a precise comprehension of their environment and clearly detect the wall and the border of the behavioral device by means of non-visual perceptions or sensory modalities. The question remains, how did they detect the wall or the border of the behavioral device, and which non-visual perceptions or sensory modalities contribute to spatial navigation in blind mice. A large body of evidence showed that early blindness (due to retinopathy of prematurity, glaucoma and cataract) results in an enhancement of the remaining non-visual modalities in humans (Van Boven et al., 2000; Beaulieu-Lefebvre et al., 2011; Renier et al., 2014), as well as in mice (Zhou et al., 2017; Touj et al., 2019, 2020, 2021a,b). All of these behavioral changes are supported by brain plasticity and reorganization (Kupers and Ptito, 2014; Chebat et al., 2020; Touj et al., 2020). Compensatory mechanisms can be adopted to improve spatial and navigation skills. For example, spatial navigation in blind individuals can be driven by other non-visual sensory cues, such as auditory, tactile or olfactory cues, supported by intramodal and crossmodal brain plasticity (Merabet and Pascual-Leone, 2010; Gori et al., 2017). In addition to the fact that mystacial vibrissae are the most important sensory organ used for exploration in mice, a possibility would be that subtle background noise in the room and sounds produced by the mice themselves can be used in echolocation to detect the border or wall of the devices (Arnott et al., 2013).

Furthermore, we evidenced that blind mice traveled 1.5-fold more than the sighted mice in the open-field, the EPM, and the FST tests. Our findings are consistent with previous studies using cone-rod homeobox (Crx) knockout mice as a mouse model of retinopathy (Iura and Udo, 2014) and late enucleated mice (Dyer and Weldon, 1975: strains from Balb/cJ, C57BL/6J, SEC/1reJ, DBA/2J, Swiss/CD) and rats (Klein and Brown, 1969). Interestingly, a study conducted on albino rats reported the effects of heightened exploration and alternation behaviors in (1) anosmic, (2) peripherally blinded, and (3) anosmic and blind rats, compared to sighted ones, in an open-field maze (Klein and Brown, 1969). Taken together, those studies highlight that sensory loss produces an increase in locomotor exploratory activity. Several hypotheses can be proposed to explain the hypermobility in our congenital blind strain. Firstly, an enhancement of the general exploratory locomotion in the light phase can be due to the endogenous circadian rhythm alteration induced by early blindness during the light/dark cycle (Iura and Udo, 2014; Ramamurthy and Krubitzer, 2018). Secondly, it is possible that blind mice compensate for their lack of visual information when exploring a novel environment by gathering enough information and memorizing them before they become familiar (Iura and Udo, 2014). This concept of “optimal level of excitation” proposed by Hebb and Thompson (1954) stipulates that, given that exploratory and curiosity drive aroused by external stimuli (Berlyne, 1955), visual deprivation may change the pattern and extent of the exploration. Blind animals may have greater exploratory activity than sighted ones, as they should travel more to achieve the same level of central arousal using their remaining sensory inputs.

Interestingly, structural MRI and histological analyses conducted on the ZRDBA blind mice showed plasticity in a multitude of sensory brain areas, such as reduced vision-related structures (superior colliculus, optic tracts, dorsal lateral geniculate nucleus, and primary visual cortex) and enhancement of the orbital, auditory, and olfactory areas (Touj et al., 2020). Additionally, regions involved in the fear and anxiety circuits (LeDoux, 2000; Etkin, 2012), including the amygdala, anterior cingulate and medial prefrontal cortices, and stria terminalis, are all found larger in congenitally blind mice. It should also be noted that some mesolimbic structures regulating the reward system such as the nucleus accumbens are also enlarged in blind ZRDBA mice. Importantly, enlargement of the structures involved in navigation and spatial memory (Rosenstock et al., 1977; Aggleton et al., 1995), including nuclei in mammillary bodies and fimbria-fornix, were evidenced in anophthalmic compared with sighted mice (Touj et al., 2020). Taken together, anatomical changes in those brain regions may contribute to reduced anxiety and hyperactivity seen in our anophthalmic mice. More generally, structural plasticity may support a compensatory mechanism allowing early blind individuals to be perfectly adapted to their environment. Future research should investigate whether structural plasticity in these brain areas may affect function and specific behaviors in this mouse model of congenital blindness.

The translation of our findings from this mouse model to early blindness in humans should be interpreted with caution. Firstly, the discrepancy reported in human and rodent studies could be due to the importance of vision, since it is the most critical and developed sense in humans whereas olfaction or tactile sensory cues are more salient in mice. Secondly, causes for congenital blindness in human studies are various, preserving the eye and visual pathways in most cases whereas the characteristic of the ZRDBA strain mice is to be born without eyes and optic nerves. Nevertheless, a recent study conducted on the ZRDBA strain found that dark-reared sighted mice from birth show similar hypersensitivity than their anophthalmic counterparts, underlying that these results applied to early visual deprivation in general and they were not specific to mice devoid of the visual system (Touj et al., 2021b). More importantly, it has been largely investigated that early visual deprivation (regardless of the etiologies) leads to dramatic behavioral and brain plasticity in humans, consistent with murine models, especially on most of sensory systems (Kupers and Ptito, 2014; Renier et al., 2014; Zhou et al., 2017; Touj et al., 2020, 2021a,b).

Several limitations of our study can be noticed. We chose to use a longitudinal approach that consists in testing the same animals in various behavioral tests. The repeated measures likely affect the behavioral responses due to increased manipulation and habituation or stress throughout the whole procedure. However, statistical corrections have been performed for multiple comparisons and we were able to examine whether the variables measured in each test were correlated between them. From a methodological point of view, weak or no depression-like behavioral responses (i.e., immobility) reported in the FST and the Suspension tail test in both sighted and blind mice, highlights the necessity of developing and validating new sensitive behavioral tests (FST and STS being mostly used in the screening of potential antidepressant drugs). Since we assessed anxiety-like behaviors based on visual and non-visual cues using the open-field test and the elevated plus maze, future studies should examine the ability to identify danger by means of behavioral paradigms based on non-visual cues only. For example, a new avenue of research would be to investigate whether early blindness impacts fear and defensive responses induced by predators' cues/signals, such as cat/fox odors or owl sounds. It is of particular interest to examine the effects of the onset of visual deprivation on the onset of depression in a mouse model to assess whether a mouse model mirrors the high prevalence of depression induced by progressive or sudden visual impairment or blindness in humans occurring later in life. Developing a mouse model allows us to modulate the onset and the duration of the visual deprivation, and to examine whether these two factors are predictors of the presence/absence and the severity of some psychological disorders.

## Conclusion

In the present study, a mouse model of congenital blindness was used to assess depression-like and anxiety-like behaviors induced by early visual deprivation. Behavioral analysis of early blind mice revealed no depressive behavior, lower anxiety associated with intensifying exploratory locomotion. This mouse model will allow exploration of underlying mechanisms of multi-modal brain plasticity supporting these behavioral adaptations.

## Data Availability Statement

The raw data supporting the conclusions of this article will be made available by the authors, without undue reservation.

## Ethics Statement

Experimental procedures and animal use were approved by the Animal Care Committee of the Université du Québec à Trois-Rivières, in accordance with the guidelines of the Canadian Council on Animal Care.

## Author Contributions

All authors listed have made a substantial, direct, and intellectual contribution to the work and approved it for publication.

## Funding

This work was funded by the Natural Sciences and Engineering Research Council of Canada (NSERC) (2017-06942).

## Conflict of Interest

The authors declare that the research was conducted in the absence of any commercial or financial relationships that could be construed as a potential conflict of interest.

## Publisher's Note

All claims expressed in this article are solely those of the authors and do not necessarily represent those of their affiliated organizations, or those of the publisher, the editors and the reviewers. Any product that may be evaluated in this article, or claim that may be made by its manufacturer, is not guaranteed or endorsed by the publisher.
